# Verifying Head Impacts Recorded by a Wearable Sensor using Video Footage in Rugby League: a Preliminary Study

**DOI:** 10.1186/s40798-019-0182-3

**Published:** 2019-03-14

**Authors:** Lauchlan Carey, Peter Stanwell, Douglas P. Terry, Andrew S. McIntosh, Shane V. Caswell, Grant L. Iverson, Andrew J. Gardner

**Affiliations:** 1 Centre for Stroke and Brain Injury, School of Health Sciences, Faculty of Health, University of Newcastle, Callaghan, New South Wales Australia; 2 Department of Physical Medicine and Rehabilitation, Harvard Medical School, Boston, MA USA; 3 Spaulding Rehabilitation Hospital, Boston, MA USA; 4 MassGeneral Hospital for Children™ Sport Concussion Program, & Home Base, A Red Sox Foundation and Massachusetts General Hospital Program, Boston, MA USA; 5 School of Engineering and Australian Collaboration for Research into Injury in Sport and its Prevention, Edith Cowan University, Perth, Western Australia Australia; 6 Monash University Accident Research Centre, Monash University, Clayton, Victoria Australia; 7 Sports Medicine Assessment Research & Testing (SMART) Laboratory, George Mason University, Manassas, Virginia USA; 8 Hunter New England Local Health District Sports Concussion Program, New Lambton Heights, New South Wales Australia; 9 Centre for Stroke and Brain Injury, School of Medicine and Public Health, University of Newcastle, Callaghan, New South Wales Australia

**Keywords:** Head impacts, Rugby league, Wearable sensors, Accelerometer, Video review

## Abstract

**Background:**

Rugby league is a full-contact collision sport with an inherent risk of concussion. Wearable instrumented technology was used to observe and characterize the level of exposure to head impacts during game play.

**Purpose:**

To verify the impacts recorded by the x-patch™ with video analysis.

**Study design:**

Observational case series.

**Methods:**

The x-patch™ was used on eight men’s semi-professional rugby league players during the 2016 Newcastle Rugby League competition (five forwards and three backs). Game day footage was recorded by a trained videographer using a single camera located at the highest midfield location to verify the impact recorded by the x-patch™. Videographic and accelerometer data were time synchronized.

**Results:**

The x-patch™ sensors recorded a total of 779 impacts ≥ 20 g during the games, of which 732 (94.0%) were verified on video. In addition, 817 impacts were identified on video that did not record an impact on the sensors. The number of video-verified impacts ≥ 20 g, per playing hour, was 7.8 for forwards and 4.8 for backs (range = 3.9–19.0). Impacts resulting in a diagnosed concussion had much greater peak linear acceleration (*M* = 76.1 g, SD = 17.0) than impacts that did not result in a concussion (*M* = 34.2g, SD = 18.0; Cohen’s *d* = 2.4).

**Conclusions:**

The vast majority (94%) of impacts ≥ 20 g captured by the x-patch™ sensor were video verified in semi-professional rugby league games. The use of a secondary source of information to verify impact events recorded by wearable sensors is beneficial in clarifying game events and exposure levels.

## Key Points

- Wearable instrumented technology has the potential to quantify the kinematic responses of the head when exposed to head impact forces during contact and collision sports.
- The vast majority of high acceleration impacts (≥ 20 g) recorded on sensor were verified on video review of the games, and impacts resulting in a medically diagnosed concussion had greater peak linear acceleration than impacts that did not result in a concussion.
- There was a substantial number of possible false-positive high-acceleration impacts recorded on the sensors before, during, and after the games. It is recommended that until sensor technology improves, head impact sensor data are used in conjunction with video.

## Background

Rugby league is a high-intensity collision sport [32] with a risk of concussive injury for participants [18]. Concussion can occur in rugby league through direct head impacts and potentially through indirect impacts, i.e., inertial or impulsive [44], because both can give rise to the necessary brain loadings considered to cause concussion. The incidence of medically diagnosed concussions in three clubs in the National Rugby League (NRL) was 14.8 and 8.9 per 1000 player match hours in 2013 [20] and 2014 [19], respectively. The incidence rate of suspected concussions based on use of the “concussion interchange rule” was 24.0 per 1000 NRL player match hours [21]. No study has reported on the incidence of concussion at semi-professional or amateur levels of competition [18]. Accurately and quickly identifying a concussion is especially important in the NRL, both for player safety and team strategy. If a player is evaluated for but not diagnosed with a concussion, the team is forced to use one of their 12 “interchanges,” while if the player is diagnosed with a concussion, an interchange is not used.

Using technology to assist in identifying head impacts and concussion, diagnosis has been increasingly common. For instance, sideline video review and analysis has been introduced in a number of professional leagues worldwide to improve the recognition of a possible concussion [40]. Several studies have reviewed the usefulness and limitations of sideline and post-game video review [15, 22, 38, 40]. For example, some studies evaluating video use in professional leagues [15, 19, 21, 22, 24, 38, 40, 41] reported that video injury surveillance can be difficult to interpret, but may provide a useful adjunct to the recognition of concussion [6]. There has also been interest in examining whether impact sensors can identify head impacts. Impact sensors have been used in a number of research studies of helmeted (e.g., American football [4–7, 11, 16, 26, 29, 52, 54, 56, 61], ice hockey [45–47, 57, 58]), and non-helmeted sports (e.g., football/soccer [30, 43], rugby union [34, 36], rugby league [37], Australian rules football [33, 55], lacrosse [49], mixed martial arts [31]) to the kinematic responses to forces applied to the head during participation in sports. The validity of these impact sensors has been examined in controlled laboratory studies [1–3, 8, 9, 14, 28, 33, 39, 51, 53, 60], suggesting peak linear acceleration as measured by the x-patch™ has reasonable agreement with the Hybrid III anthropomorphic test device (ATD) head-neck system, but the angular velocity measured by the the x-patch™ had much poorer agreement. The low sampling frequency of the x-patch™ has been suggested to be a reason for the poor agreement [48]. Although numerous studies recorded the total number of impacts that occurred while players wore the sensors [27, 28, 31–33, 35, 40, 46, 55], few of the studies verified those impacts via video [31, 49, 59] or were not able to differentiate direct head impacts from indirect impacts.

A growing body of research has combined the use of impact sensors with game video to verify the accuracy of impacts recorded by this wearable technology. However, a more complete assessment of the validity of all impacts using additional sources of information is required [12, 50]. For example, only 16% of recorded impacts were verified on video in a study of women’s collegiate football (soccer) [50], 65% in boys high school lacrosse [12], and 32% in girls high school lacrosse [10], suggesting that the wearable sensor technology may substantially overestimate impact events [13]. Few studies have examined rugby league using these technological advances. Gardner and colleagues [21] reviewed video footage of concussions and suspected concussions in the NRL. They reported that 98% of initial (primary) impacts occurred to the head/face, but they did not localize the impact location any further. More recently, King and colleagues [37] reported a total of 1977 impacts in 88 h of game play during a single season of junior rugby league using the x-patch™ sensor, with 48% of impacts reported to have occurred to the side of the head, 26% to the front of the head, and 25% to the back of the head. However, every event (> 10 g) captured by the x-patch™ sensor will be deemed to be a direct head impact and assigned a location on the head, regardless of whether such an impact occurred. No previous studies have combined video analysis and impact data from sensors in rugby league. The primary aim of this study was to determine the reliability of x-patch™-derived measurements of head impact exposures. A secondary aim was to describe the playing characteristics and game play situation of the video-verified impacts in semi-professional men’s rugby league.

## Methods

### Participants

Data were prospectively collected from a men’s semi-professional rugby league team during the 2016 Newcastle Rugby League season. A total of 8 players (mean age 25.5 years, SD 4.7 years) from a single club consented to participate. The participant’s playing position consisted of five forwards and three backs. A typical rugby league team formation involves 13 players (7 backs, 5 forwards) on the field at one time. During the course of the season, there were six medically diagnosed concussions in four players. Concussion was diagnosed by a medical practitioner. The operational diagnosis was consistent with the Concussion in Sport Group (Berlin, 2016) [42] definition. The research protocol was approved by the University of Newcastle Human Research Ethics Committee (reference no. H-2015-0323). The study was also endorsed by the Newcastle Rugby League and the participating club.

### Measures

#### Impact Sensors

All consenting participants wore x-patch™ sensors (X2 Biosystems) during all games that they participated in during the 2016 Newcastle Rugby League season. The x-patch™ sensors were attached to the skin covering the right mastoid process of each player because previous literature has suggested that sensor positioning over the mastoid process is crucial to ensure that it is not activated by soft tissue effects during impacts [60]. Each sensor was uniquely labeled and applied to the players by a trained member of the research team in the change rooms. Sensors were affixed and activated before the team’s warm-up, approximately 30 min before the beginning of the game. An alcohol wipe was used to clean the skin behind the ear over the right mastoid process before a Convacare protective barrier wipe (ConvaTec Inc.) was applied to help with adhesion of the area. The sensor was attached using a double-sided adhesive patch and worn for the entire game.

The x-patch™ contains a triaxial accelerometer and gyroscope that measure linear and angular kinematics. These are applied to estimate the head’s kinematic responses, e.g., resultant linear acceleration and peak linear acceleration (PLA), peak rotational velocity and acceleration (PRV), and location of impact. The slope of the relationship between the actual PLA in a laboratory setting and the PLA measured by the x-patch™ has been reported as 0.972, which is similar to the expected relationship of 1.0 (*p* = .14) [53]. However, the relationship between the actual peak angular acceleration (PAA) and the measured (PAA) was only 0.7745 (*p* = .0027), which is statistically different from the expected 1.0 relationship. Therefore, we decided to not report in detail the angular head kinematic data (i.e., angular velocity and angular acceleration). The x-patch™ does not measure impact force and cannot differentiate between a direct head impact and an impact to another part of the body that results in acceleration of the head, i.e., impulsive or inertial loading. The x-patch™ records data when linear acceleration exceeds 10 g; at which point, the x-patch™ saves 10 ms prior to the impact and 90 ms after, with a maximum of 1000 data points per channel. During the study, each x-patch™ was removed immediately after each game in the change room, and data were downloaded to the Injury Management Software (IMS; X2Biosystems). The IMS produces a time-stamped line output for each “impact” which included PLA, PRA, PAV, HIC, and other variables. The x-patch™ does not measure impact force, and therefore, any perturbation of the wearer that causes a linear head acceleration greater than 10 g is recorded as a “head impact,” even when direct head impact did not occur. The sensors were cleared of data before being charged and stored for the next game.

#### Video Review

Each game was digitally recorded by a trained videographer. One single-view high-definition camera was positioned at the highest possible vantage point at the field’s midline (i.e., the center). Close-up shots panned left and right following the ball to maximize the visibility of game play, players, and potential impacts. Game footage was analyzed in conjunction with data obtained from the x-patch™. Video time was synchronized with time stamps from the sensors using the first three impacts from each player. Each half of the game was viewed from start to finish using QuickTime X (Apple Inc.) by one author (LC). The reviewer has experience in watching professional rugby league. Video was played back at an appropriate speed to verify whether or not an impact occurred. This consisted of pausing, replaying, and using slow motion as needed. Every impact on video was matched to the sensor data for validation. An impact was defined as “any contact, to the head or trunk/torso, made to the player wearing an x-patch™ by another player or the playing surface.” Sensor-recorded impacts not verified with video were identified. Similarly, impacts on video that did not correlate with x-patch™-recorded impacts were identified. Impacts were categorized as either direct (defined as an impact that made direct contact with the head) or indirect (defined as an inertial or impulsive impact from contact made to the body rather than directly to the head). Impacts were also characterized by play characteristics (i.e., *attacking—*running with the ball, *defending*—tackling, and *off-the-ball incidents*—no player was in possession of the ball when contact between players was made). They were also characterized into number of tacklers (i.e., 1–4), wrestling impacts that happen after the initial contact (yes/no), side of contact (i.e., left, right, back, or front on), area of contact (i.e., head, shoulder, chest, arm, waist, or below), and whether an impact appeared to be direct contact with the sensor (yes/no). The type of game-play scenario was also considered. As previously described in the video review of rugby league game play [22], the “hit-up” was defined as “a type of play where the ball carrier charges directly into an organized defensive line.” Data were coded in Microsoft Excel.

### Statistical Analyses

Descriptive statistics (i.e., frequencies, percentages, medians, IQRs, and standard deviations) of peak linear acceleration (PLA) and peak rotational velocity (PRV) for all verified head impacts ≥ 20 g were calculated by player position and game-play situation. Similar to criteria applied in previous studies [10, 43], the review of impacts was limited to ≥ 20 g to remove low acceleration events (< 20 g) commonly associated with physical activities of game play (e.g., jumping, hard stops, sharp changes of direction) and unlikely to result in deleterious neurophysiological changes. Impact rates per player game hours (PGH) with corresponding 95% confidence intervals (CIs) were constructed. The impact rate was calculated as the number of verified impacts divided by the number of PGH. The formula for calculating the impact rate is provided below.

$$ \mathrm{Impact}\ \mathrm{Rate}=\frac{\Sigma \mathrm{verified}\ \mathrm{impacts}\ge 20\mathrm{g}}{\Sigma \mathrm{PGH}} $$

Players engaged in game play for different amounts of time over the course of the season. We calculated verified impacts per minute. Cross tabulations were conducted between the impact sensors’ estimated head impact location and the characterization conducted using video analysis. Data were also reviewed for playing position (i.e., forward versus back) and play characteristics (i.e., attacking, defending, off-the-ball). An exploratory *t* test was used to compare the rate that forwards and backs sustained verified impacts. Exploratory analyses compared the acceleration (i.e., PLA) between verified/non-verified impacts, direct/indirect impacts, and concussion/non-concussion impacts using non-parametric tests because PLA was not normally distributed. The Mann-Whitney (MW) test was used when there was homogeneity of variances, and the Kolmogorov-Smirnov (KS) test was used when variances were unequal. All analyses were performed using SPSS 23 (IBM Corp.).

## Results

### Impact Frequency

During the 2016 Newcastle Rugby League competition, eight participants were instrumented with the wearable sensors and game video was captured during all games. These athletes played a total of 91 games across the season (89.1 player game hours/5346 player game minutes). Stratified by playing position, data from forwards accounted for 39.6% (2117 min) and data from backs accounted for 60.4% (3229 min) of playing time. The x-patch™ became detached from each player at least once during the season, for a total of 183 min of lost data due to detached sensors for the season (backs *M* = 19 min, SD = 16.46, range = 15–50; forwards *M* = 12 min, SD = 19.38, range = 2–51). In addition, there was a 135-min game time lost due to faulty sensors (all forwards).

**Fig. 1:**
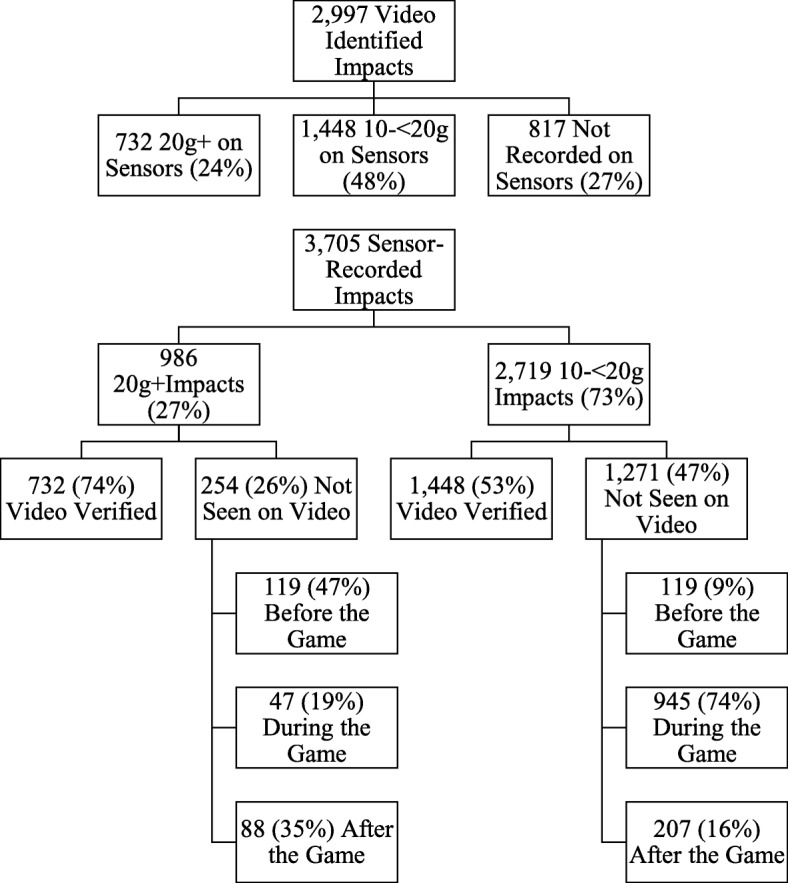
Flow diagram comparison of video-identified impacts and sensor-recorded impacts

There were 2997 *video-verified* impacts to the eight players (Table 1). Of those, 732 were recorded as 20 g or greater (24%), 1448 video-verified direct and indirect impacts were recorded as between 10 g and 20 g (48%), and 817 (27%; 36 direct vs 781 indirect) were not recorded on the sensors (see Fig. 1). The video review revealed 36 direct head impacts (24 to the side of the head, 6 to the front, and 6 to the back) that did not result in any data being recorded on the x-patch™, in addition to 21 impacts (registered as > 30 g) recorded by the x-patch™ that were not verified on video. There was no significant difference in the PLA of video-verified versus non-video-verified impacts (verified *M* = 34.5, SD = 18.3; non-verified *M* = 41.2, SD = 29.2; KS *Z* = 0.96, *p* = .32; Cohen’s *d* = − 0.27).

**Table 1:** Frequency of video-verified impacts by game time and playing position

		Video-verified		Sensor-recorded 10 g+		Sensor-recorded 10 g to < 20 g		Sensor-recorded ≥ 20 g		Sensor-recorded and video-verified ≥ 20 g		Sensor-recorded and video-verified 10 g to < 20 g	
	Playing hours (min)	Game impacts (*n*)	Impacts per game hour (n)	Game impacts (*n*)	Impacts per game hour (*n*)	Game impacts (*n*)	Impacts per game hour (*n*)	Game impacts (*n*)	Impacts per game hour (*n*)	Game impacts (*n*)	Impacts per game hour (*n*)	Game impacts (*n*)	Impacts per game hour (*n*)
1st half	44.6 (2673)	1545	34.6	1632	36.6	1249	28.0	383	8.6	370	8.3	768	17.2
2nd half	44.6 (2673)	1452	32.6	1540	34.5	1144	25.7	396	8.9	362	8.1	680	15.2
Forwards	35.3 (2117)	1716	48.6	1323	37.5	902	25.6	421	11.9	407	11.5	798	22.6
Backs	53.8 (3229)	1281	23.8	1849	34.4	1491	27.7	358	6.7	325	6.0	650	12.1
Total sample	89.1 (5346)	2997	33.6	3172	35.6	2393	26.9	779	8.7	732	8.2	1448	16.3

There were 3705 *sensor-recorded* impacts to the 8 players (see Fig. 1). Of those, 1525 (41.2%) were not seen on video. Of the 1525 impacts that were not seen on video, 533 (35.0%) occurred before or after the game. Interestingly, there were 254 impacts registered as ≥ 20 g that were not seen on video, and of those, 119 occurred before the game (47%, presumably during warm up), 47 occurred during the game (19%), and 88 occurred after the game (35%). It seems particularly unusual that there would be 88 ≥ 20 g impacts and 207 10 to 20 g impacts to these 8 players occurring after the game had ended.

### Direct Head Impacts by Game and Player Characteristics

Of the 732 video-verified impacts, 536 were identified as direct head impacts. Of the 536 video-verified direct head impacts, the ball carrier (attacker) recorded 261 (48.7%) and the tackler (defender) recorded 253 (47.2%), while 22 were recorded during an off-the-ball incident (4.1%; incidental contact *n* = 9; contact with the playing surface *n* = 4; melee/scuffle or fighting *n* = 6; contact celebrating tries *n* = 3). The number of impacts recorded in the first half (49.3%) compared to the second half of games (50.7%) was similar. Players sustained an average of 0.10 direct impacts per minute played (SD = 0.07, range = 0.04–0.27), which equates to an average of one verified impact per 10.0 min. Forwards sustained more verified impacts per minute compared to backs (forwards *M* = 0.13, SD = 0.08, 1 per 7.2 min; backs *M* = 0.08, SD = 0.03, 1 per 13.3 min). An exploratory independent sample *t* test suggests that forwards and backs did not statistically differ in the rate they sustained direct impacts (*t*(7) = 1.04, *p* = .33). However, effect size analysis shows a large, meaningful difference between the rates they sustained impacts based on playing position (Cohen’s *d* = 0.83). The individual player data by playing position, game time impact, and video verification are provided in Table 2.

**Table 2:** Cross tabulation of frequency of verified in-game impacts measured by the x-patch™, video-verified impacts, player position, and game time

	Playing position	Player time in game (min)	Sensor-recorded in-game impacts	Video-verified game impacts	Percentage of impacts verified	Video-verified impacts per game hour	Video-verified direct impacts	Video-verified direct impacts per game hour
Player 1	Forward	365	29	29	100	4.8	15	2.5
	Back	687	62	53	85.5	4.6	41	3.6
Player 2	Forward	259	63	62	98.4	14.3	27	6.3
Player 3	Back	1120	73	73	100	3.9	49	2.6
Player 4	Forward	568	182	180	98.9	19.0	155	16.4
Player 5	Back	808	126	105	83.3	7.8	85	6.3
Player 6	Forward	505	74	68	91.9	8.1	44	5.2
Player 7	Back	614	97	94	96.9	9.2	67	6.5
Player 8	Forward	420	73	68	93.2	9.7	53	7.6
Total		5346	779	732	94.0	8.2	536	6.0

Season totals. Player 1 played in both forward and back positions during the season. Sensor-recorded impacts were ≥ 20gs

### Impact Mechanism and Location

When stratifying all video-verified direct head impacts (*n* = 536) by the location of impact from the x-patch™, the most impacts occurred to the front (*n* = 190, 35.4%) and side (*n* = 194, 36.2%), with fewer to the back (*n* = 121, 21.3%) and top (*n* = 31, 5.3%) of the head. When examining the location of the verified impacts on video review, the most impacts were determined to occur to the side (*n* = 498, 92.9%) and front (*n* = 27, 5.0%%), with fewer impacts to the back (*n* = 11, 2.1%). The x-patch™ accurately recorded the location of video-verified direct head impacts in 55.6% of video-verified impacts to the front of the head, 38.0% of video-verified impacts to the side of the head, and 54.5% of video-verified impacts to the back of the head. The sensor recorded 39 impacts to the top of the head, whereas no impact was verified by video review as impacting the top of the head (see Table 3).

**Table 3:** Video-verified impacts: location accuracy of direct and indirect impacts

	Total				Direct impact				Indirect impact			
	x-patch™ (*n*)	Video (*n*)	Agreement (*n*)	Accuracy (%)	x-patch™ (*n*)	Video (*n*)	Agreement (*n*)	Accuracy (*%*)	x-patch™ (*n*)	Video (*n*)	Agreement (*n*)	Accuracy (%)
Front	273	134	56	41.8	190	27	15	55.6	83	107	41	38.3
Side	264	582	228	39.2	194	498	189	38.0	70	84	39	46.4
Back	156	16	7	43.8	121	11	6	54.5	35	5	1	20
Top	39	0	0	0	31	0	0	0	8	0	0	0
Total	732	732	291	39.8	536	536	210	39.3	196	196	81	41.3

Direct impacts (as determined by video review) had a greater PLA compared to indirect impacts (direct *M* = 37.59, SD = 20.09; indirect *M* = 26.22, SD = 7.67; KS *Z* = 4.14, *p* < .001, *d* = 0.75), as well as greater PRV compared to indirect impacts (direct *M* = 27.70, SD = 11.55; indirect *M* = 21.97, SD = 9.02; KS *Z* = 2.77, *p* < .001, *d* = 0.55). Secondary impacts during a tackle (i.e., impacts after the initial contact) accounted for 261 (35.7%) of total impacts (Table 4). There were 580 tackles that resulted in the 732 video-verified impacts. For 480 tackles, there was 1 impact recorded; for 83, there were 2 impacts recorded; for 15, there were 3 impacts recorded; and for 2, there were 4 impacts recorded. There were additional 32 tackles occurring off the ball that resulted in 33 video-verified impacts (31 with 1 impact recorded and 1 with 2 impacts). The hit-up was a play that accounted for approximately 52% (*n* = 301) of all x-patch™ recorded events. Of the hit-up plays, forward positions accounted for 44% (*n* = 132) of those impacts, while backs accounted for approximately 56% (*n* = 169) of impacts. A summary of the total impacts per play and the total impacts per play for forward and back positions is provided in Table 4.

**Table 4:** Total x-patch™-recorded impacts per play

Impact-recorded type of play	One hit-up	One tackle	Total	Two hit-up	Two tackle	Total	Three hit-up	Three tackle	Total	Four hit-up	Four tackle	Total	One off the ball	Two off the ball	Total
Week 1	26 (10, 16)	15 (7, 8)	41 (17, 24)	7 (4, 3)	1 (1, 0)	8 (5,3)	0 (0,0)	0 (0,0)	0 (0,0)	0 (0,0)	0 (0,0)	0 (0,0)	2 (1,1)	0 (0,0)	2 (1,1)
Week 2	18 (8,10)	17 (15,2)	35 (23,12)	1 (0,1)	1 (1,0)	2 (1,1)	0 (0,0)	0 (0,0)	0 (0,0)	0 (0,0)	0 (0,0)	0 (0,0)	1 (0,1)	0 (0,0)	1 (0,1)
Week 3	9 (5,4)	30 (23,7)	39 (28,11)	1 (1,0)	2 (0,2)	3 (1,2)	0 (0,0)	0 (0,0)	0 (0,0)	0 (0,0)	0 (0,0)	0 (0,0)	1 (1,0)	1	2 (1,1)
Week 4	24 (11,13)	32 (28,4)	56 (39,17)	4 (2,2)	6 (5,1)	10 (7,3)	0 (0,0)	1 (1,0)	1 (1,0)	0 (0,0)	0 (0,0)	0 (0,0)	2 (0,2)	0 (0,0)	2 (0,2)
Week 5	11 (6,5)	15 (11,4)	26 (17,9)	5 (3,2)	2 (2,0)	7 (5,2)	1 (1,0)	1 (0,1)	2 (1,1)	2 (2,0)	0 (0,0)	2 (2,0)	2 (0,2)	0 (0,0)	2 (0,2)
Week 6	22 (10,12)	30 (19,11)	52 (29,23)	6 (2,4)	8 (6,2)	14 (8,2)	0 (0,0)	1 (1,0)	1 (1,0)	0 (0,0)	0 (0,0)	0 (0,0)	4 (2,2)	0 (0,0)	4 (2,2)
Week 7	14 (7,7)	16 (15,1)	30 (22,8)	4 (2,2)	1 (1,0)	5 (3,2)	1 (0,1)	0 (0,0)	1 (0,1)	0 (0,0)	0 (0,0)	0 (0,0)	3 (1,2)	0 (0,0)	3 (1,2)
Week 8	28 (17,11)	7 (4,3)	35 (21,14)	2 (1,1)	2 (2,0)	4 (3,1)	3 (1,2)	1 (1,0)	4 (2,2)	0 (0,0)	0 (0,0)	0 (0,0)	3 (0,3)	0 (0,0)	3 (0,3)
Week 9	10 (5,5)	14 (5,9)	24 (10,14)	4 (1,3)	1 (1,0)	5 (2,3)	1 (0,1)	0 (0,0)	1 (0,1)	0 (0,0)	0 (0,0)	0 (0,0)	2 (0,2)	0 (0,0)	2 (0,2)
Week 10	9 (1,8)	4 (1,3)	13 (2,11)	2 (0,2)	0 (0,0)	2 (0,2)	0 (0,0)	0 (0,0)	0 (0,0)	0 (0,0)	0 (0,0)	0 (0,0)	2 (0,2)	0 (0,0)	2 (0,2)
Week 11	13 (1,12)	5 (3,2)	18 (4,14)	2 (0,2)	1 (0,1)	3 (0,3)	0 (0,0)	0 (0,0)	0 (0,0)	0 (0,0)	0 (0,0)	0 (0,0)	3 (0,3)	0 (0,0)	3 (0,3)
Week 12	20 (10,10)	7 (4,3)	27 (14,13)	3 (1,2)	2 (0,2)	5 (1,4)	1 (0,1)	0 (0,0)	1 (0,1)	0 (0,0)	0 (0,0)	0 (0,0)	1 (0,1)	0 (0,0)	1 (0,1)
Week 13	9 (4,5)	10 (3,7)	29 (7,22)	0 (0,0)	0 (0,0)	0 (0,0)	1 (1,0)	0 (0,0)	1 (1,0)	0 (0,0)	0 (0,0)	0 (0,0)	2 (1,1)	0 (0,0)	2 (1.1)
Week 14	7 (4,3)	15 (13,2)	22 (17,5)	2 (1,1)	5 (5,0)	7 (6,1)	0 (0,0)	1 (0,1)	1 (0,1)	0 (0,0)	0 (0,0)	0 (0,0)	2 (1,1)	0 (0,0)	2 (1,1)
Week 15	13 (3,10)	13 (12,1)	26 (15,11)	3 (2,1)	3 (2,1)	6 (4,2)	0 (0,0)	0 (0,0)	0 (0,0)	0 (0,0)	0 (0,0)	0 (0,0)	0 (0,0)	0 (0,0)	0 (0,0)
Week 16	8 (3,5)	9 (9,0)	17 (12,5)	2 (1,1)	0 (0,0)	2 (1,1)	2 (2,0)	0 (0,0)	2 (2,0)	0 (0,0)	0 (0,0)	0 (0,0)	1 (1,0)	0 (0,0)	1 (1,0)
Total	241 (105,136)	239 (172,67)	480 (277,203)	48 (21,27)	35 (26,9)	83 (47,36)	10 (5,5)	5 (3,2)	15 (8,7)	2 (1,1)	0 (0,0)	2 (1,1)	31 (8,23)	1 (0,1)	32 (8,24)
Average/week	15.1 (6.6,8.5)	14.9 (10.8,4.2)	30 (15.4,12.7)	3 (1.3,1.7)	2.2 (1.6,0.6)	5.2 (2.9,2.3)	0.6 (0.3,0.3)	0.3 (0.2,0.1)	0.9 (0.5,0.4)	0.1 (0.1,0.1)	0 (0,0)	0.1 (0.1,0.1)	1.9 (0.5,1.4)	0 (0,0.1)	2 (0.5,1.5)

Data in the parentheses are for forwards and backs, as follows: (forwards, backs)

### Diagnosed Concussions

There were six diagnosed concussions during the season. All six concussions (100%) occurred as a result of a direct head impact. The PLA of the impacts that resulted in a diagnosed concussion was much greater (*M* = 76.1g, SD = 17.02, range = 61.6–106.6 g) than video-verified direct impacts that did not result in a concussion that were > 20 g (*M* = 34.20 g, SD = 17.96; MW *U* = 183.50, *p* < .001, *d* = 2.39). Figure 1 shows the PLA and PRV for all video-verified impacts with the six concussions highlighted.

## Discussion

**Fig. 2:**
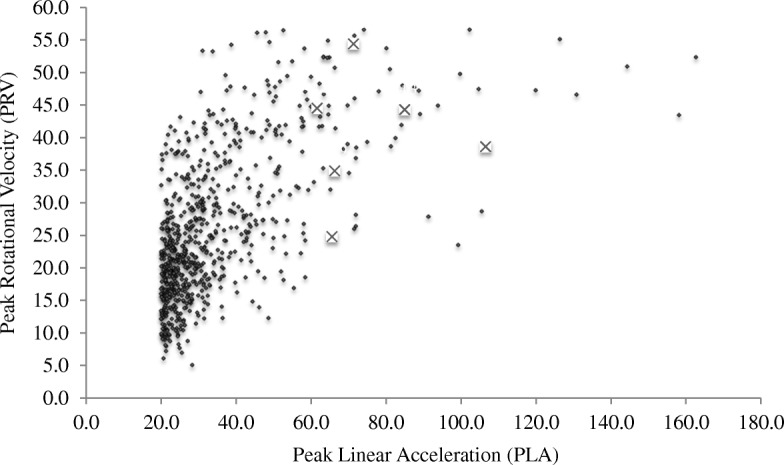
Scatterplot of video-verified impacts recorded by the x-patch™. X: medically diagnosed concussion impacts

The purpose of this study was to verify the information recorded by wearable impact sensors to determine the reliability of the data, in addition to describing the playing characteristics and game-play situation of the video-verified impacts in semi-professional men’s rugby league. Eight semi-professional male rugby league players wore wearable impact sensors during a single season. The vast majority of high acceleration impacts during games were verified on video. Specifically, there were 779 in-game sensor-recorded impacts ≥20 g, of which 732 were verified on video (i.e., 94%; Table 2). Differences in the number of video-verified impacts per hour of playing time were observed based on playing position, such that forwards had greater exposure compared to backs, consistent with previous literature [17, 25]. As seen in Fig. 2, impacts resulting in a medically diagnosed concussion had much greater peak linear acceleration (*M* = 76.1 g, SD = 17.0) than impacts that did not result in a concussion (*M* = 34.2 g, SD = 18.0; *d* = 2.39). However, as seen in Fig. 1, there were six (0.82%) video-verified direct head impacts above the highest recorded concussion PLA, and 53 (7.2%) video-verified direct head impacts above the lowest recorded concussion PLA, that did not result in concussion—suggesting a host of factors beyond the impact acceleration may contribute to acute neurological disturbances of concussion.

There were 817 tackles or impacts on video review that did not result in any recording at all on the impact sensor (i.e., false negatives). It is difficult to determine the true level of false-negative data because video review does not enable determination of the observed impacts to the head that occur above or below the 10 g threshold (i.e., which impacts are at a sub-threshold and do not register on the x-patch™). In addition, given that for 101 (17%) tackles, there was more than 1 impact (i.e., in a single tackle, multiple impacts were recorded) registered by the x-patch™, the true level of false-negative data could also be much greater than the 817 because on video review, it was only the initial impact that was considered as being missed, and not any subsequent impact that may have been recorded during the same tackle.

Our reported rate of video-verified true sensor-detected impacts may be an overestimate, and it might be confounded by player position. Rugby league is a demanding physical contact game with frequent body contact. Defensive players and “hit-up” players (i.e., forwards) may have a greater body contact rate than backs. If the x-patch™ records spurious head impact events, as appeared to be the case before and after match play, by chance, the video review is likely to identify an impact event (i.e., some body contact) when the x-patch™ records a spurious head impact. Rugby league may not be the ideal setting for validating all characteristics of head impact sensors. The rate of false-positive sensor-detected impacts cannot be determined. There were 295 impacts recorded on the sensors after the game had ended. Of those, 88 were ≥ 20 g impacts. It seems unusual that there would be so many large impacts recorded after the game, and we cannot determine the extent to which the sensors yield false-positive findings before, during, or after the game. In general, the rate of false-positive impacts, in addition to the possibility that the device could register greater linear acceleration than was actually the case, *raises concern that impact sensor studies that do not verify impacts may be over-reporting both the level of exposure of athletes* (i.e., *the number of true head impacts*) *and the severity of the head impacts* (i.e., *the magnitude of the head’s kinematic responses*).

On average, players sustained 1 verified impact every 10.0 min. The rate that these athletes sustained impacts appeared to differ based on their playing position, with forwards sustaining impacts more frequently than backs. Our exploratory (and underpowered) independent sample *t* test did not show group differences, but this non-significant finding is likely an artifact of the small sample size in our pilot study; effect size analysis showed a large, meaningful difference in the number of impacts stratified by playing position (Cohen’s *d* = 1.15). We have previously reported that the concussion interchange rule (a rule used by club medical staff to remove and assess a player suspected of having sustained a concussion to determine the player’s suitability to remain in a game or be permanently removed) was used more commonly in forwards than in backs at the professional level (forwards 57%, backs 43%) [21] and the national youth level (forwards 66%, backs 34%) [22]. In the current study of semi-professional rugby league players, the impact exposure levels (as measured by the x-patch™ and verified on video) revealed the forward positions are more commonly exposed to contact with the head during match play than the backline positions.

On-field play characteristics may also be associated with head impacts and potential concussions. A prior study showed that the concussion interchange rule was used to remove the tackler 55% and 61% of the time compared to 43% and 38% for the ball carrier at the professional and national youth level, respectively [22]. In the current study, approximately 44% of the video-verified impacts involved the tackler, approximately 51% involved the ball carrier, and approximately 5% were recorded during an off-the-ball incident. A tackler making an upper body tackle high on the ball carrier making a hit-up was the most common play leading to the use of the concussion interchange rule, accounting for 26% of all uses at the professional level [21]. In the current study of semi-professional rugby league players, the hit-up play accounted for approximately 49% (*n* = 301) of all verified x-patch™-recorded impacts, with the tackler accounting for 46% (*n* = 279) and 5% (*n* = 32) coming from off-the-ball incidents, a similar result as the professional level. In addition, the hit-up play data revealed that forward positions accounted for 44% (*n* = 132) and backs approximately 56% (*n* = 169) of impacts recorded.

The current study offers some insights into the level of impacts sustained in the sport of rugby league at the semi-professional level. Although the results are preliminary given the small sample size, it is clear that there appears to be variations in impact exposure by position (forwards versus backs), type of play (i.e., the hit-up), game situation (ball carrier versus tackler), and number of tacklers involved in a tackle. These findings also offer further insights into the previous rugby league video analysis studies that have been conducted at the professional level [19–23] examining risks for activation of a head injury assessment (HIA) and subsequent diagnosed concussion.

In the current study six concussions were diagnosed in players wearing the x-patch™. There was a large, statistically significant difference between the PLA of concussive impacts versus non-concussive impacts. Future studies should aim to collect a larger number of concussion events in order to calculate the possible thresholds of PLA (as well as other parameters) and sensitivity and specificity of those impact thresholds to determine the potential clinical application of the x-patch™ or similar devices for assisting in the clinical diagnosis of concussion.

There are a few limitations to the current study design. First, using only eight players is the small sample size and limits the generalizability of the findings. Second, the false-negative incidences only considered a single (i.e., initial) impact as missing, as such, the possibility that a second or subsequent impact registration was also not coded by the impact sensor was not calculated as a “missed impact” resulting in an underestimation of the potential false negatives. Third, each video was coded by a single researcher. It is possible that some impacts were missed by the researcher when reviewing video. Fourth, the current findings may not be generalizable to all levels of rugby league or other sports. Fifth, the current findings are limited to the x-patch™ impact sensor only and may not reflect the capabilities of other impact sensors. Finally, there is no accepted standard as to what head impact-related PLA represents a “subconcussive” hit (e.g., 10 to 20 g) apart from a strict clinical outcome, nor is it understood how or whether these impacts affect brain microstructure or function. Future studies with larger sample sizes may consider reviewing the role cumulative impacts may have on the vulnerability of an athlete to future concussive events. In addition, in view of the literature that suggests that cumulative head impact exposure is a predisposing factor for the onset of concussion [27, 55], reviewing the potential increased vulnerability for subsequent concussions in athletes who sustain multiple impacts and multiple concussions may also be an important focus in future larger studies. It may also be useful for future studies to extend video recordings to before and after the match so that non-match play head impacts can be better understood.

## Conclusion

These data are consistent with the findings from Cortes and colleagues [13] who found that using a secondary source of information to verify head impact events recorded by wearable sensors was beneficial in clarifying game events. Similar to those boys and girls lacrosse players [13], a considerable number of false-positive head impacts were recorded by the wearable sensors in our semi-professional rugby league players. This illustrates the value of adding an additional source of information (i.e., video) when quantifying the impacts players sustain. The implementation of a standard verification method could assist in validating data and reducing false-positive rates. It is important that these false-positive results are described appropriately in impact sensor studies to more accurately communicate the findings of impact sensor research [13]and better brain loading patterns, and inform scientists/industry leaders about the limitations of current wearable technology. Such findings have practical implications for how impact sensors should be used and how existing data should be interpreted. Video confirmation of impacts and time synchronization can support a more accurate measure of impact frequency/magnitude and characterization of head impact events [13]. The use of a secondary source to verify impacts will assist in clarifying the severity of the impacts and the level of burden of exposure for an athlete.

## Abbreviations

ATD: Anthropomorphic test device
CI: Confidence interval
HIA: Head injury assessment
KS: Kolmogorov-Smirnov
M: Mean
MW: Mann-Whitney
NRL: National Rugby League
PGH: Player game hours
PLA: Peak linear acceleration
PRV: Peak rotational velocity
SD: Standard deviation
TM: Trademark
